# Chromatin accessibility reveals insights into androgen receptor activation and transcriptional specificity

**DOI:** 10.1186/gb-2012-13-10-r88

**Published:** 2012-10-03

**Authors:** Alok K Tewari, Galip Gürkan Yardimci, Yoichiro Shibata, Nathan C Sheffield, Lingyun Song, Barry S Taylor, Stoyan G Georgiev, Gerhard A Coetzee, Uwe Ohler, Terrence S Furey, Gregory E Crawford, Phillip G Febbo

**Affiliations:** 1 Institute for Genome Sciences & Policy, Duke University, Durham, NC 27708, USA; 2 Helen Diller Family Comprehensive Cancer Center, University of California at San Francisco, San Francisco, CA 94115, USA; 3 Computational Biology Center, Memorial Sloan-Kettering Cancer Center, New York, NY 10065, USA; 4 Department of Preventive Medicine, Norris Cancer Center, Keck School of Medicine, University of Southern California, Los Angeles, CA 90089, USA; 5 Department of Urology, Norris Cancer Center, Keck School of Medicine, University of Southern California, Los Angeles, CA 90089, USA; 6 Department of Biostatistics and Bioinformatics, Duke University, Durham, NC 27708, USA; 7 Department of Computer Science, Duke University, Durham, NC 27708, USA; 8 Departments of Biology and Genetics, Carolina Center for Genome Sciences and Lineberger Comprehensive Cancer Center, University of North Carolina at Chapel Hill, Chapel Hill, NC 27599, USA; 9 Department of Pediatrics, Division of Medical Genetics, Duke University, Durham, NC 27708, USA; 10 Department of Medicine, University of California at San Francisco School of Medicine, San Francisco, CA 94115, USA; 11 Department of Urology, University of California at San Francisco School of Medicine, San Francisco, CA 94115, USA

## Abstract

**Background:**

Epigenetic mechanisms such as chromatin accessibility impact transcription factor binding to DNA and transcriptional specificity. The androgen receptor (AR), a master regulator of the male phenotype and prostate cancer pathogenesis, acts primarily through ligand-activated transcription of target genes. Although several determinants of AR transcriptional specificity have been elucidated, our understanding of the interplay between chromatin accessibility and AR function remains incomplete.

**Results:**

We used deep sequencing to assess chromatin structure via DNase I hypersensitivity and mRNA abundance, and paired these datasets with three independent AR ChIP-seq datasets. Our analysis revealed qualitative and quantitative differences in chromatin accessibility that corresponded to both AR binding and an enrichment of motifs for potential collaborating factors, one of which was identified as SP1. These quantitative differences were significantly associated with AR-regulated mRNA transcription across the genome. Base-pair resolution of the DNase I cleavage profile revealed three distinct footprinting patterns associated with the AR-DNA interaction, suggesting multiple modes of AR interaction with the genome.

**Conclusions:**

In contrast with other DNA-binding factors, AR binding to the genome does not only target regions that are accessible to DNase I cleavage prior to hormone induction. AR binding is invariably associated with an increase in chromatin accessibility and, consequently, changes in gene expression. Furthermore, we present the first *in vivo *evidence that a significant fraction of AR binds only to half of the full AR DNA motif. These findings indicate a dynamic quantitative relationship between chromatin structure and AR-DNA binding that impacts AR transcriptional specificity.

## Background

The androgen receptor (AR), a ligand-activated member of the nuclear receptor superfamily, plays a critical role in the male phenotype and prostate cancer biology. AR expression results in context-specific transformation of prostate epithelial cells [1-5], and persistent AR signaling is implicated in the progression to castration-resistant prostate cancer [6-8]. However, AR activity can be alternatively associated with promotion or inhibition of growth. For example, AR activation by androgen induction limits proliferation in some immortalized prostate epithelial cells expressing AR [2,9], whereas AR activation most often increases proliferation in human-derived prostate cancer cell lines with endogenous AR expression (for example, LNCaP [10], LAPC-4 [11] and VCaP [12]). As the AR acts primarily through transcriptional activation of target genes, it is critical to understand the determinants of the AR-mediated transcriptional program.

AR-mediated transcriptional specificity is highly regulated, and the AR associates with proteins that possess co-activator or co-repressor function [13]. AR binding to chromatin, similar to many transcription factors, is thought to occur in competition with nucleosome histone proteins, the core organizational component of chromatin [14]. Several identified AR co-factors either possess an intrinsic chromatin remodeling capability or are able to bind and recruit other chromatin modifying enzymes and facilitate AR binding. Indeed, the binding of AR to DNA across the genome (the AR cistrome) is modulated by the primary DNA sequence, chromatin structure around the AR and/or co-factor binding sites and other factors such as FOXA1, a member of the forkhead box (FOX) and hepatocyte nuclear factor transcription factor families [15].

Recent reports examining nucleosome positioning in relation to AR binding have found that local nucleosome depletion and increased chromatin accessibility accompanies the AR binding to DNA [16,17]. However, while one study observed a clear decrease in occupancy of histone 3 (H3) dimethyl lysine 4 (H3K4me2)-marked nucleosomes over AR binding sites and a concomitant increase in occupancy at flanking nucleosome positions [16], another found that the nucleosome depletion size was not increased by AR occupancy but rather nucleosome dynamics were affected by the receptor binding [17]. Interestingly, nucleosome depletion at the three enhancers studied was evident both before and after hormone treatment. Thus, chromatin structure is likely to impact the interaction between the AR and DNA, and ligand activation of the AR may result in altered chromatin structure. Our complete understanding of this process remains quite limited and, consequently, a comprehensive genome-wide analysis of AR function is needed.

The mapping of DNase I hypersensitive (DHS) sites is an accurate method to identify different types of active gene regulatory elements within accessible chromatin [18-20]. More recent high-throughput identification of all DHS sites within a single cell type using DNase-seq show high correlations with active histone modifications, regions of nucleosome depletion detected by Formaldehyde Assisted Isolation of Regulatory Elements (FAIRE) and transcription factor binding sites [21-24]. Changes in DNase I cleavage patterns have been observed at specific loci bound by nuclear receptors, supporting the finding that at least some nuclear receptors can disrupt chromatin structure [25]. The single-base-pair resolution digestion patterns from DNase-seq can identify footprints of local DNA protection that accurately predict transcription factor-DNA binding [26-28]. Thus, in a single experiment, DNase-seq can identify both larger nucleosome-depleted regions and finer resolution transcription factor binding sites within nucleosome-depleted regions.

To determine the relationship between AR-dependent chromatin accessibility changes and AR-mediated transcription, we performed DNase-seq and mRNA-seq on the well-established androgen-sensitive prostate cancer cell line LNCaP, before and after hormone induction. AR binding sites were obtained from three published studies describing AR ChIP-seq experiments on LNCaP cell lines. Another member of the nuclear receptor superfamily, the glucocorticoid receptor (GR), was recently found to bind predominantly in DHS sites that exist prior to GR ligand hormone treatment [29]. In agreement with another recently published study [30], we find that a substantial amount of AR binding occurs in accessible chromatin after hormone induction. In contrast to the GR, approximately half of these AR sites bind in DHS sites that exist prior to AR activation, with the remaining sites becoming accessible after AR activation. AR binding also significantly increases chromatin accessibility. Quantitative changes in chromatin structure correlate with AR-dependent differential gene expression and are enriched for transcription factor-DNA binding motifs that offer insight into the mechanism of AR-induced chromatin remodeling. Intriguingly, fine resolution DNase-seq profiles surrounding AR DNA binding motifs provides *in vivo *evidence of AR binding to both half and full AR DNA recognition motifs. Together, our work reveals that active chromatin remodeling occurs during androgen nuclear receptor activation.

## Results

### DNase-seq identifies changes in chromatin accessibility with androgen receptor activation

**Figure 1:**
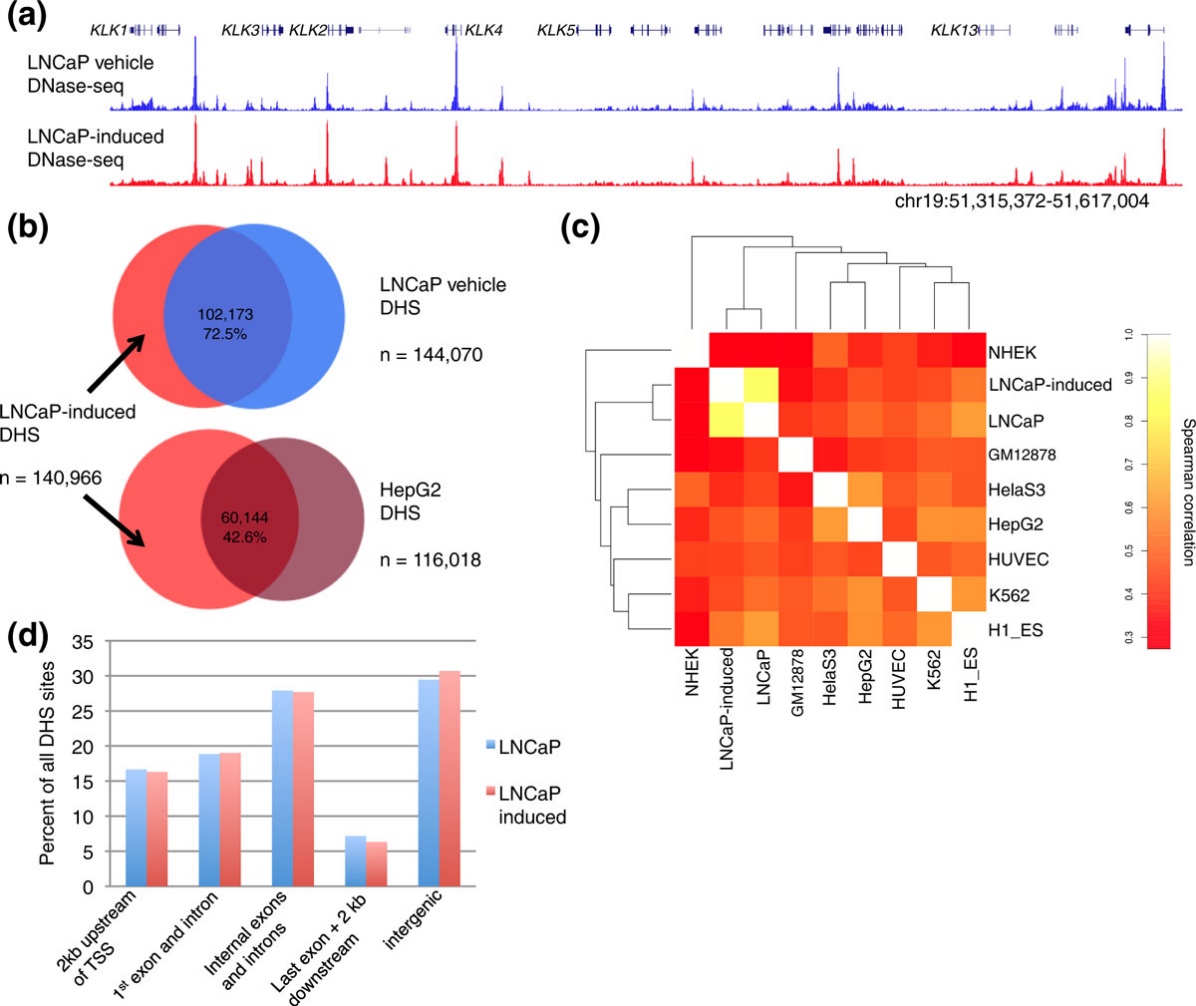
**Identification of DNase I hypersensitive sites in LNCaP cells before and after R1881 stimulation**. **(a) **DNase-seq signal is a continuous signal across the genome. We illustrate the chromatin accessibility around the KLK locus before and after hormone induction. Each sample has a fixed y-axis DNase-seq score of 0.7. **(b) **Overlap between DHS sites identified before and after hormone as compared to the unrelated cell line HepG2. **(c) **Spearman correlation heatmap of the union set of top 100,000 DHS peaks in each of the nine cell lines illustrated. **(d) **Distribution of all DHS sites relative to genic elements. DHS: DNase I hypersensitive; DNase-seq: DNase I hypersensitivity analysis coupled with high-throughput sequencing; kb; kilobase pairs; TSS: transcription start site.

To assess the relationship between accessible chromatin and AR activation, we performed DNase-seq on independent growths of LNCaP cells that were cultured with (LNCaP-induced) or without (LNCaP) the synthetic androgen R1881 (12 hours). Using previously published methodologies and a standard analysis pipeline [31], we identified the full spectrum of DNase-seq signal across the genome (Figure 1a). We approached interpretation of DNase-seq data in two ways: calling discrete peaks, referred to as DHS sites, and comparing regions qualitatively as binary conditions (DHS site or not); and identifying regions of statistically different DNase-seq signal before and after hormone treatment, referred to as ΔDNase regions.

From approximately 130 million post-filter sequence reads per growth condition, we identified 144,070 DHS sites in LNCaP and 140,966 DHS in LNCaP-induced cells using a *P*-value cutoff of 0.05. The DHS sites in each cell condition cover approximately 3% of the human genome (Table S1 in Additional file 1). A comparison of the DHS sites identified in LNCaP-induced and LNCaP reveals that 102,173 (72.5%) of sites overlap. To put the degree of overlap in context, we used the same criteria to identify DHS sites in seven unrelated cell lines for which high quality DNase-seq data is available (NHEK, GM12678, HelaS3, HepG2, HUVEC, K562 and H1-ES) [24]. The average overlap between distinct cell lines is 50.4% ± 7.04%, which is substantially less than the overlap between LNCaP and LNCaP-induced (Figure 1b,c). We also investigated the overall distribution of DHS sites relative to genic elements and found that AR activation does not shift this distribution (Figure 1d). These data suggest that although AR activation induces a modest amount of chromatin changes, the degree of these changes is substantially less than those detected between cell lines from unrelated tissues.

**Figure 2:**
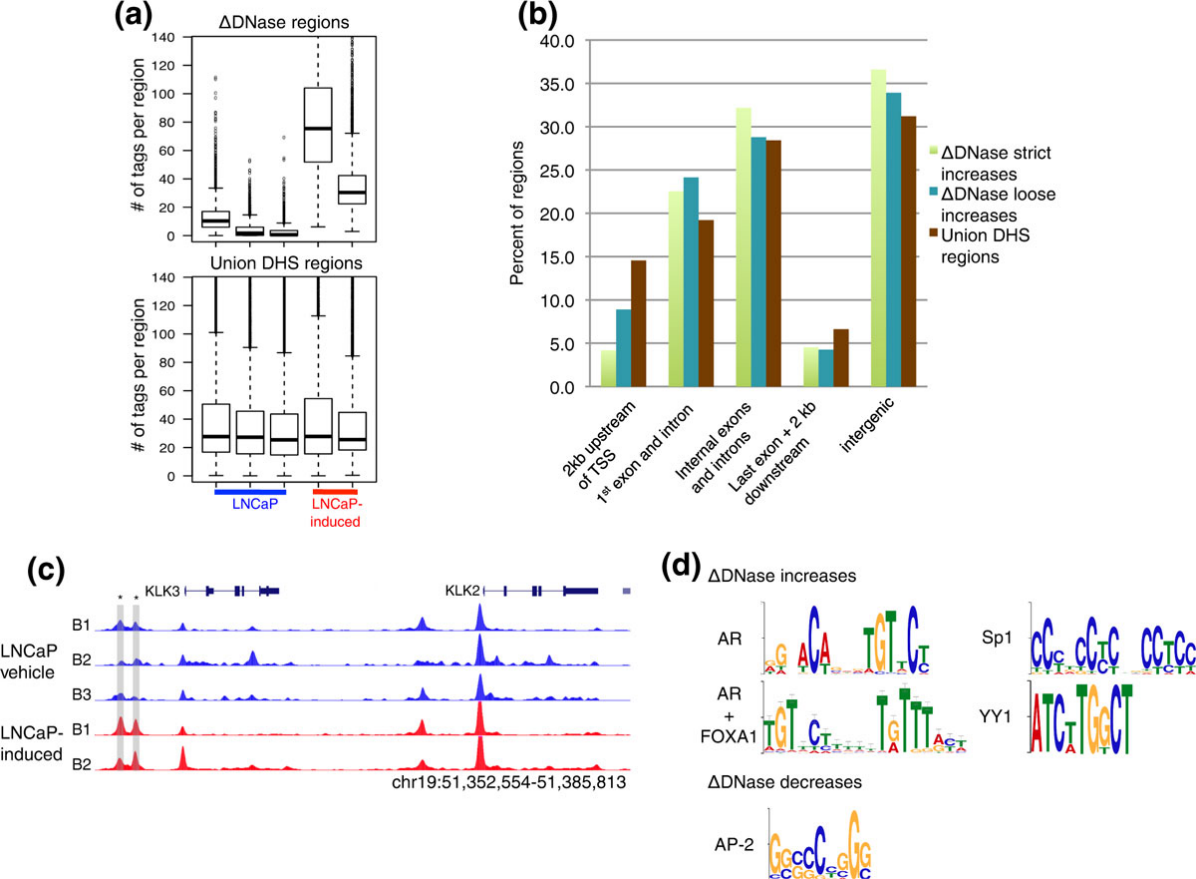
**Identification of differences in DNase-seq signal**. **(a) **Top panel: distribution of tags per ΔDNase windows in LNCaP versus LNCaP-induced. Bottom panel: distribution of DNase-seq tags in union regions used to identify ΔDNase increases and decreases. **(b) **Distribution of ΔDNase regions and all union (of LNCaP and LNCaP-induced) DHS regions relative to genic elements. **(c) **Replicates of DNase-seq data around *KLK3 *and *KLK2*. Y-axis is fixed to range from 0 to 0.4 for all rows. Highlighted regions marked by an asterisk represent examples of significant ΔDNase increases. **(d) **Significant motifs identified *de novo *in ΔDNase gain and loss regions. DHS: DNase I hypersensitive; DNase-seq: DNase I hypersensitivity analysis coupled with high-throughput sequencing; kb; kilobase pairs; TSS: transcription start site.

To quantitatively identify those loci with the most substantive increase or decrease in DNase-seq signal with AR activation, we used the edgeR statistical package [32]. Increases represent regions that become more accessible after hormone treatment, and decreases become less accessible. To capture a broad spectrum of significant changes in signal, we used two statistical thresholds (strict = a false discovery rate (FDR) threshold of 5%, and loose = unadjusted *P*-value threshold of 0.05) to identify the degree of accessibility changes, which we refer to as ΔDNase regions. At the strict threshold, we identified 2,586 regions with strict ΔDNase increase after androgen induction and no regions of signal decrease. The loose threshold identified 18,692 regions with loose ΔDNase increase and 1,467 regions with loose ΔDNase decrease (Table 1). These regions suggest that AR activation results primarily in regions with increased rather than decreased chromatin accessibility (Figure 2a, Figure S1A in Additional file 1).

**Table 1:** Number of differential regions of DNase-seq with androgen receptor activation (ΔDNase).

Strict threshold	Number of regions
Strict ΔDNase increase	2,586
Strict ΔDNase decrease	0

**Loose threshold**	

Loose ΔDNase increase	18,692
Loose ΔDNase decrease	1,467

ΔDNase regions were identified using edgeR (Materials and methods). Strict ΔDNase increases are a complete subset of the loose ΔDNase increase regions.

To ensure that the observed trend towards higher levels of open chromatin is not a bias related to the edgeR algorithm, we also calculated a normalized differential DNase-seq tag count for each region in the union set of LNCaP-induced and LNCaP DHS sites (Materials and methods). This differential count also indicated that more regions display an increase in DNase-seq signal with androgen treatment, supporting the edgeR results (Figure S1B,C in Additional file 1).

Mapping all regions of significantly changed DNase-seq signal to genic elements revealed a depletion of promoter regions and enrichment for both inter- and intragenic locations compared with all DHS sites (Figure 2b, Figure S1D in Additional file 1). For example, approximately 8% of loose ΔDNase increases map to promoters (defined as 2 kb region upstream of the transcriptional start site) whereas close to 15% of all DHS sites fell within promoters. The opposite trend was seen for sites that overlapped the first exon and/or intron and sites contained within intergenic regions. Thus, our data show that AR activation primarily results in increased chromatin accessibility in distal regulatory elements that may be associated with enhancer rather than proximal promoter function, exemplified by a well-characterized AR enhancer [33] 4 kb upstream of the kallikrein 3 (*KLK3*) promoter (Figure 2c).

We hypothesized that ΔDNase regions represented locations where AR activation altered transcription factor binding. As expected, we found a strong AR motif match in regions of increased open chromatin (Materials and methods). In addition, several other significantly enriched motifs were detected in both ΔDNase increase and decrease regions (Figure 2d, Table S2 in Additional file 1) that correspond to transcription factors such as specificity protein 1 (SP1). We also detected enrichment of an SP1 DNA recognition motif within DHS sites using a self-organizing map (SOM) (Materials and methods) that identifies highly specific LNCaP-only DHS regions that were not accessible in 113 additional cell lines (Figure S1E in Additional file 1, top panel). The SOM analysis also identified an enriched motif corresponding to E2A/TCF3 as well as FOXA1 (Figure S1E, middle panel and bottom panels). SP1 can bind directly with multiple known AR co-factors as well as the AR [34]. TCF3 is involved in the Wnt/β-catenin signaling pathway, which crosstalks with AR signaling in prostate cancer [35]. ΔDNase increases were also enriched for a yin and yang 1 (YY1) motif, which is a transcription factor with a known role in AR-mediated transcription [36]. To compare how often these motifs are found in ΔDNase regions relative to other DHS sites, we calculated a relative enrichment score reflecting the relative frequency that a motif match is found in either set of regions. The score for the AR (4.82) and AR+FOXA1 (2.36) motifs suggests they are more commonly found in ΔDNase regions, whereas the score for SP1 (0.45) suggests that this motif is more commonly found in regions in which chromatin accessibility does not change with AR activation. The score for YY1 (1.05) indicates that the motif is found with almost equal frequency in ΔDNase regions and other DHS sites. ΔDNase regions that underwent a decrease in chromatin accessibility with AR activation did not exhibit an enrichment of the AR motif, but we uncovered a motif consistent with activator protein 2, which has been implicated in estrogen receptor binding and function [37], and its DNA motif is found in the promoter regions of several AR-regulated genes in prostate cancer [38]. Thus, AR activation changes chromatin accessibility in regions with AR and AR co-factor binding motifs, likely due to changes in transcription factor loading at these genomic regions.

### The androgen receptor binds both poised and remodeled chromatin accessible to DNase I cleavage

Based on our motif analysis of ΔDNase regions and recent reports of AR binding to nucleosome-depleted regions marked by acetylated H3 [17] and H3K4me2 [16], we hypothesized that the AR binds primarily in DHS sites. We therefore used three sets of AR ChIP-seq data from LNCaP cells (Table 2) that we refer to as Yu [39], Massie [40] and Coetzee [17,41]. To minimize the impact of technical variation within each individual experiment, we created two high confidence sets of AR binding sites from these three sources: an 'R1881 intersect' set consisting of Yu and Massie peaks that overlap each other, as these experiments used the same AR hormone ligand as our DNase-seq experiments (R1881); and an 'All AR Intersect' dataset containing the intersection of peaks from all three data sets including the Coetzee experiment that used an alternative AR ligand, dihydrotestosterone. Intersecting AR ChIP datasets did not change the distribution of identified binding sites relative to genic elements (Figure S2A in Additional file 1), despite substantially reducing the number of total AR binding regions by including only those common to two or more experiments (Table 2).

**Table 2:** Characteristics of androgen receptor ChIP-seq datasets.

Dataset	Number of androgen receptor binding sites
Massie	19,505
Yu	37,676
Coetzee	12,929
R1881 intersect (Massie/Yu intersect)	13,258
All AR Intersect	5,940

Number of peaks called for each AR ChIP-seq dataset. R1881 intersect represents the intersection of the Massie and Yu datasets. All AR Intersect represents high confidence androgen receptor binding sites that are found in all three datasets.

**Figure 3:**
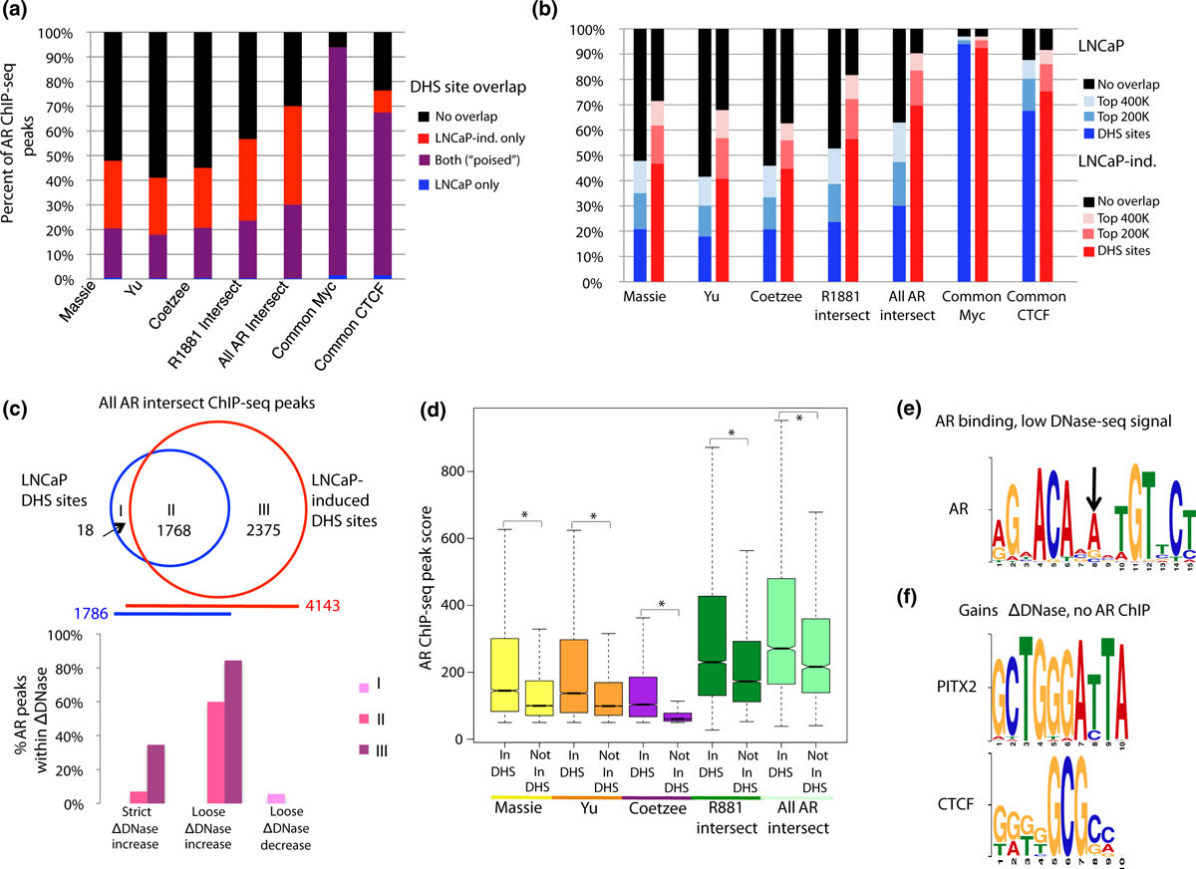
**Relationship between androgen receptor binding and DNase I hypersensitivity**. **(a) **Overlap of each ChIP-seq AR binding peaks with poised LNCaP DHS (regions that are DHS sites in both LNCaP and LNCaP-induced) and LNCaP-induced only DHS sites. AR binding sites not overlapping a DHS site are represented in black. Common Myc and CTCF binding sites are shown as control. **(b) **Overlap of ChIP-seq peaks is shown at different thresholds of DNase-seq enrichment ('DHS sites' representing regions of significant signal over background *P *< 0.05, 'Top 200k' representing the top 200,000 initial peaks showing enrichment over background, and 'Top 400k' representing all regions showing DNase-seq enrichment over background). Columns in various shades of blue show overlap with LNCaP DHS at different thresholds, and columns in various shades of red show overlap with LNCaP-induced DHS at different thresholds. Common Myc and CTCF binding sites [24] are included as control. **(c) **Overlap between ΔDNase regions and AR binding sites in the context of AR binding sites that overlap with DHS sites. Shown are data for All AR ChIP-seq intersect peaks. Region I represents AR binding sites in LNCaP DHS sites only, Region II contains AR binding sites in a region that is both a LNCaP DHS site and LNCaP-induced DHS site (poised), and Region III represents AR binding sites in a region that is only a LNCaP-induced DHS site. Bottom figure shows overlap with ΔDNase strict and loose gain as well as loose decreases. Each region of overlap (I, II, III) is indicated by a different shade of purple. **(d) **AR ChIP-seq binding scores for peaks overlapping and not overlapping DHS sites as measured by MACS. Starred data points denote significant differences in AR peak score (Mann-Whitney *P*-value < 0.001). **(e) ***De novo *motif analysis of regions containing an AR ChIP-seq peak (All AR Intersect) and very low DNase-seq signal (black bars in Figure 3B) reveals a motif closely matching that of the AR, with a noticeable variation in the typically degenerate region (black arrow). **(F) ***De novo *motifs identified in ΔDNase regions that do not overlap AR ChIP-seq peaks (All AR Intersect). AR: androgen receptor; CTCF: CCCTC-binding factor; DHS: DNase I hypersensitive; DNase-seq: DNase I hypersensitivity analysis coupled with high-throughput sequencing.

Each of the three individual AR ChIP studies displayed consistent overlap patterns with DHS sites. In each individual experiment approximately 20% of all AR binding sites occurred within DHS sites that are present both before and after hormone treatment (poised DHS sites). An additional 20% to 30% of AR binding sites overlapped DHS sites following androgen induction. Thus, results from each dataset suggest that slightly less than half of all AR binding sites in DHS regions are poised (Figure 3a,b) and the remainder change in response to androgen treatment. The high confidence AR (R1881 intersect and All AR Intersect) binding sites displayed a similar trend. Of note, only 1% to 2% of AR binding sites map within a DHS site present in LNCaP but not LNCaP-induced cells. The amount of AR binding to both poised and LNCaP-induced DHS sites (Figure S2B in Additional file 1) is in stark contrast to Myc and CCCTC-binding factor (CTCF) binding sites [24] that almost exclusively bind within poised DHS sites (Figure 3a). Thus, of the AR binding events occurring within a DHS site, approximately half occurred in poised regions, with the majority binding to regions that displayed qualitative AR-induced chromatin remodeling.

Given the observation that a substantial number of AR binding sites occur within LNCaP-induced only DHS sites, we examined the association between AR binding events and quantitative chromatin remodeling. To test this, we evaluated AR sites that overlapped regions with increased DNase-seq signal (strict and loose ΔDNase increases). As expected, AR ChIP-seq peaks identified only within LNCaP-induced DHS sites (Region III, Figure 3c) show significant overlap with ΔDNase increase regions. Interestingly, AR binding sites in peaks found in both LNCaP and LNCaP-induced cells (Region II, Figure 3c) were also enriched for ΔDNase increases, although not to the same extent as those sites that mapped only within LNCaP-induced DHS sites. The proportions of AR binding regions that mapped to poised, LNCaP-induced DHS sites only and to ΔDNase regions were consistent across each AR binding data set (Figure S2C in Additional file 1). Analogously, we found that 36.5% of strict ΔDNase increases and 16.7% of loose ΔDNase increases overlapped the high confidence AR binding set (All AR Intersect) (Figure S2D in Additional file 1). These observations indicate that although AR binding occurred within DNA in a poised open chromatin state, a substantial increase in chromatin accessibility occurred in many of these regions after AR activation. This highlights the utility of identifying regions of ΔDNase signal in addition to regions that simply cross the binary threshold of becoming a DHS site with androgen induction. These findings support similar observations at three previously identified poised AR enhancers [17] and suggest that AR binding more globally stabilizes DHS, allowing for more DNase I cleavage after hormone treatment.

A large percentage of AR binding sites detected by each of the individual AR ChIP-seq datasets (approximately 50%) did not overlap DHS sites. To determine if this is due to a peak-calling threshold, we decreased the stringency threshold for identifying DHS sites to either the top 200,000 or top 400,000 DNase I sensitive regions. Overlap with AR ChIP-seq indicates that the proportion of AR binding sites binding in a poised versus qualitatively remodeled region was consistent regardless of the threshold, and that a substantial proportion of AR binding occurs in non-DNase I sensitive regions of the genome even after relaxing the DHS peak thresholds (Figure 3b). Even after increasing the sequencing depth two-fold, which increased the overall overlap of DHS sites with high confidence AR binding sites, 40% of these binding sites remained only within an induced DHS site (data not shown). In addition, the AR binding signal was stronger in regions overlapping DHS sites than non-DHS regions (Figure 3d), and was the strongest for AR sites common to two or three experiments. Thus, it appears that AR binding occurs at sites with a range of DNase I sensitivity and DNase I sensitivity correlates with AR binding strength.

**Figure 4:**
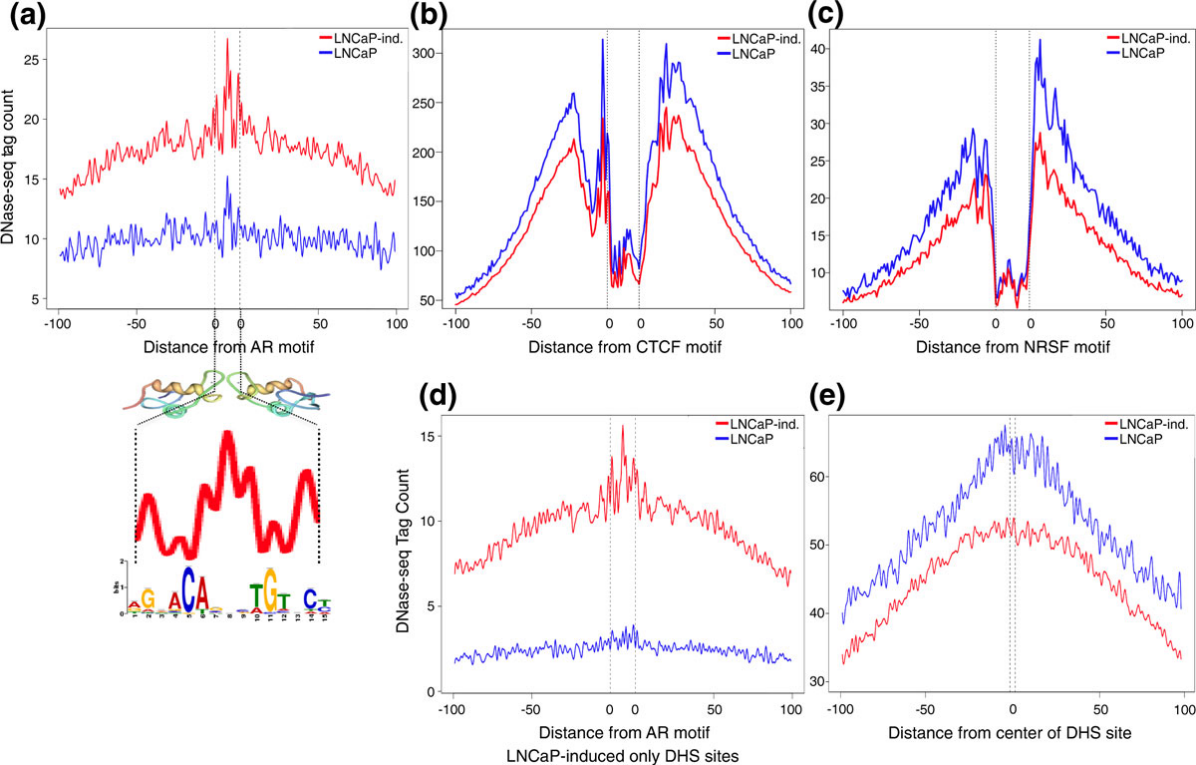
**Base-pair resolution around androgen receptor motif matches reveals a unique pattern of protection by the androgen receptor**. **(a) **Aggregate plot of DNase-seq signal around AR motif matches within poised DHS sites that also bind the AR. The pattern of DNase I cuts within the motif closely follows the known structure of the AR dimer as well as the information content of the AR DNA recognition motif determined *de novo *from ChIP-seq sequences that overlap DHS sites. **(b) **Aggregate DNase-seq signal centered around CTCF motif and **(c) **neuron-restrictive silencer factor (NRSF) matches genome-wide displaying a structurally different footprint from that of the AR. **(d) **Aggregate plot of DNase-seq signal around AR motif matches within DHS sites unique to LNCaP-induced cells that also bind the AR. **(e) **Aggregate plot of DNase-seq signal around the centers of 10,000 randomly sampled DHS sites shared between LNCaP and LNCaP-induced cells. Note that overall the aggregate signal is higher in LNCaP as compared to LNCaP-induced cells within all DHS sites. AR: androgen receptor; CTCF: CCCTC-binding factor; DHS: DNase I hypersensitive; DNase-seq: DNase I hypersensitivity analysis coupled with high-throughput sequencing; NRSF: neuron-restrictive silencer factor.

Finally, we examined several different combinations of regions for evidence of differential co-factor requirements using *de novo *motif analysis. First, we searched for motifs enriched in AR binding sites defined by ChIP-seq peaks that did not map within DHS sites. Only one motif was enriched within these regions by our *de novo *analysis, which resembled both the canonical AR motif and a motif derived *de novo *from AR ChIP-seq sequences that fall within poised DHS sites (Figure 4a), but displayed an increased invariant nucleotide within the degenerate 3 bp region between half sites (Figure 3e). Scanning these same regions against annotated motifs revealed enrichment, albeit with lower match scores, of motifs commonly associated with AR binding (Table S3 in Additional file 1). These results suggest that AR binding in regions of very low DNase-seq signal may be less associated with AR co-factors. We separated strict and loose ΔDNase increase regions into regions with and without an AR ChIP-seq peak (from All AR Intersect set) and searched for enriched motifs *de novo*. ΔDNase regions overlapping AR binding were enriched for motifs matching the AR and FOX family members, as expected. ΔDNase regions without AR binding were enriched for several high information content motifs including those for paired-like homeodomain transcription factor 2 (PITX2) and CTCF (Figure 3f, Table S3 in Additional file 1).

### Changes in chromatin accessibility correlate with the androgen receptor transcriptional program

**Figure 5:**
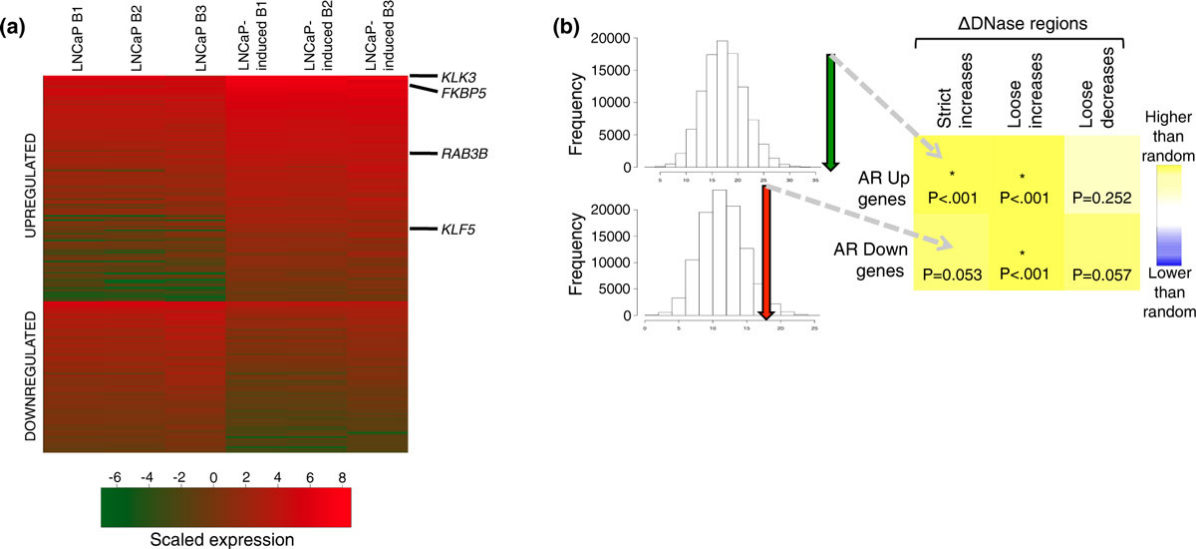
**ΔDNase regions are associated with androgen receptor-regulated transcription**. **(a) **Heatmap of mRNA-seq expression levels (natural log of reads per kilobase mapped expression value) for genes identified as differentially regulated by the AR. Rows are ordered by total sum. Genes most commonly identified in microarray studies as AR-regulated are all located near the top of the heatmap, indicating overall high levels of expression before and after hormone induction. **(b) **ΔDNase changes randomly permuted against mRNA-seq identified up- and downregulated genes. ΔDNase regions were mapped to the closest gene, and the amount of overlap between these genes and the differentially expressed set was permuted 100,000 times to assess significance. Arrows indicate the actual overlap between ΔDNase nearest genes and mRNA-seq regulated genes relative to random permutations. Blue shading represents less ΔDNase regions (absence/depletion) around regulated genes than expected by chance. Yellow shading represents more ΔDNase regions (presence/enrichment) present around regulated genes than expected by chance. AR: androgen receptor; mRNA-seq: messenger RNA abundance measured by high-throughput sequencing.

To compare ΔDNase regions to the AR-mediated transcriptional program, we generated mRNA-seq data under conditions matched to our DNase-seq experiments and identified genes differentially regulated by androgen induction. Three replicates were generated and their expression values clustered according to hormone treatment status (Figure S3A in Additional file 1). Using edgeR [32], we identified 339 genes differentially expressed upon AR induction (FDR < 0.05), 202 of which were upregulated and 137 of which were downregulated (Figure 5a, Additional file 2). Of these, 46% were identified as AR target genes in at least one other study (Additional file 3).

We hypothesized that AR-mediated changes in chromatin accessibility contribute to the AR-mediated gene expression program. By mapping ΔDNase regions to the closest transcriptional start site (Figure 5b), we found that strict ΔDNase increase regions were significantly enriched near upregulated genes (*P *< 0.001). Loose ΔDNase increases were significantly enriched near both up- and downregulated genes (*P *< 0.001). We noticed that both strict ΔDNase increases and loose ΔDNase decreases were enriched near downregulated genes with borderline significance. The reverse comparison, in which we associated differentially regulated genes to ΔDNase regions within 20 kb of the transcriptional start site (Figure S3C in Additional file 1), confirmed the strongly significant trends mentioned. By contrast, the borderline significant associations disappeared in this reverse comparison, and also when we limited our analysis from Figure 5b to a distance cutoff of 25 kb. We performed an identical analysis using ΔDNase regions and microarray expression data from Massie *et al. *[40], and observed similar associations (Figure S3B,D in Additional file 1). We also examined the association between AR binding events with very low DNase-seq signal and AR-regulated genes, and found these regions were not significantly enriched around either up- or downregulated genes (data not shown). Overall, our data support the hypothesis that AR activation preferentially causes distal increases in chromatin accessibility that significantly correlate with nearby gene expression changes.

### Base-pair resolution analysis of DNase-seq reveals multiple signal profiles

Our group and others have shown that DNase-seq can detect individual transcription factor binding events via the identification of DNase I footprints and that DNase I footprints correspond to local protection of DNA from nuclease cleavage by bound transcription factors [26-28]. An overall increase in DNase signal was observed around AR motifs (Figure 4a) compared with other transcription factor motifs such as CTCF and neuron-restrictive silencer factor (NRSF) (Figures 4b,c). A symmetrical depletion of DNase-seq signal was detected around AR motifs in DHS sites that closely matches the information content of the AR binding motif dimer (Figure 4a, red line) [42]. In poised AR binding sites, we observed a similar pattern of protection despite lower overall DNase-seq signal intensity (Figure 4a, blue line). Binding sites that became available only after androgen induction only exhibited the footprint after androgen treatment (Figure 4d, blue line). Importantly, the overall enrichment of DNase signal in LNCaP-induced cells is specific to DHS regions that bind the AR and have an AR motif, as opposed to all DHS sites (Figure 4e). The observed evidence of AR motif protection prior to androgen induction (Figure 4a) may represent binding of an alternate factor that is displaced upon AR activation, such as has been reported for specific loci by GATA binding protein 2 [17]. From the compendium of cell lines that have been processed for DNase-seq through the ENCODE project, we identified H1 embryonic stem cells and D721 medulloblastoma cells as having relatively low expression levels of the AR. DNase-seq signal around AR motifs within DHS sites in these two cell lines resemble that of LNCaP cells prior to hormone treatment (Figure S4A in Additional file 1), suggesting that such a protection pattern in non-AR activated cell lines could result from alternative transcription factor binding to DNA at these regions.

**Figure 6:**
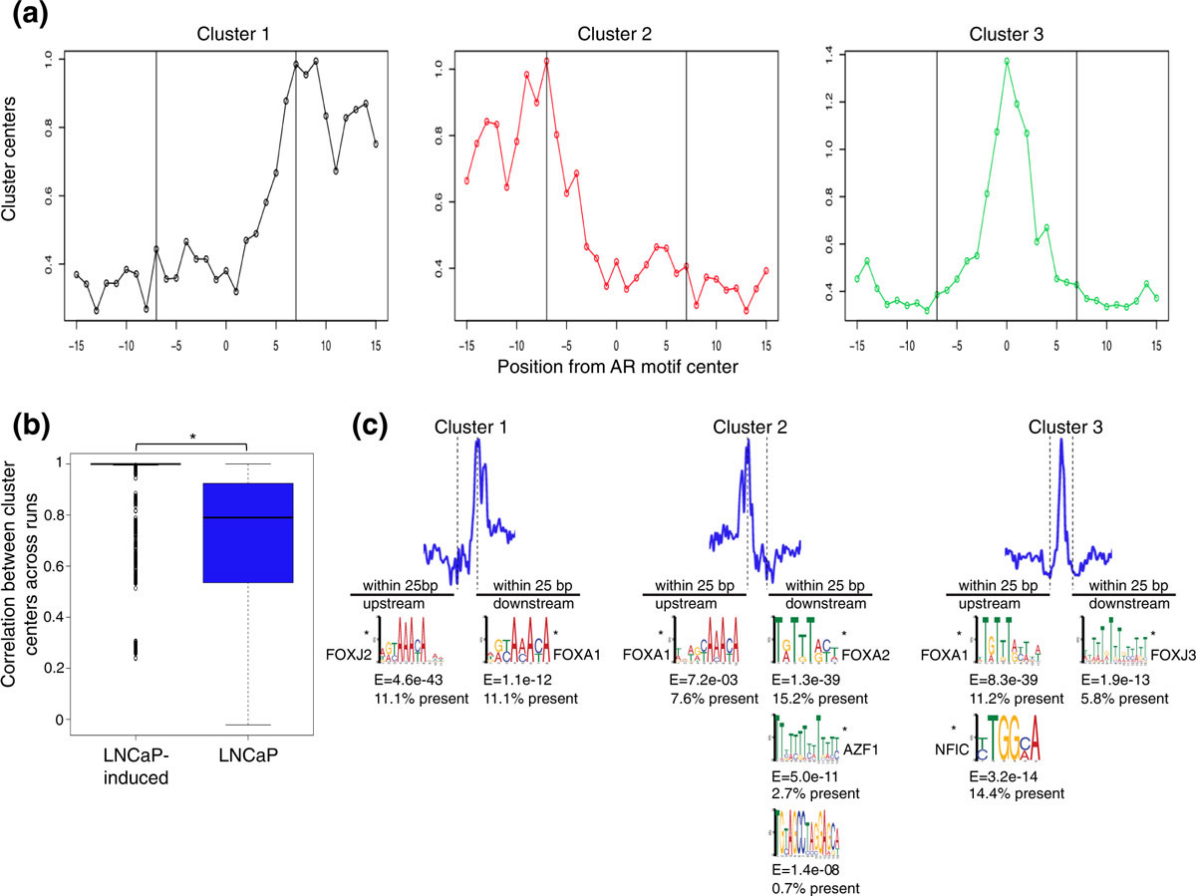
**AR binding displays three distinct modes of androgen receptor-DNA interaction that are specific to ligand-activated androgen receptor**. **(a) **K-means clustering of LNCaP-induced DNase-seq signal into three consistent clusters within AR binding sites. **(b) **K-means clustering (k = 3) was repeated 100 times on both LNCaP and LNCaP-induced DNase-seq data around all DHS sites with a full-site canonical AR motif. Shown is the distribution of correlations between cluster centers for each run. The asterisk denotes the statistically significant difference between the correlation distributions (Mann-Whitney *P *< 2.2e^-16^). **(c) **Motif analysis of the entire 25 bp span up- and downstream from AR motif matches for each cluster. MEME motifs identified within this interval (E < 0.1, E-value shown below logo) are shown in logo format. Motifs that significantly match a known motif (E < 0.05, by TomTom) are marked with an asterisk. The name of the most significant match according to TomTom is indicated next to the logo, as is the percentage of regions that contain the enriched motif. For matches resembling FOX family factors, we note that these motifs are very similar to each other. DNase-seq signal is shown as the aggregate signal from all cluster members with the dotted lines marking the location of the AR motif within the plot. AR: androgen receptor; bp: base pairs; DHS: DNase I hypersensitive; DNase-seq: DNase I hypersensitivity analysis coupled with high-throughput sequencing; FOX: Forkhead box; NF1C: nuclear factor 1 C-type.

To further investigate the AR footprint we performed k-means clustering to search for discrete DNase-seq patterns around AR motif matches (Materials and methods). DNase-seq signal was represented by a vector of DNase I cuts spanning 15 bp around the center of the AR motif. We identified three reproducible clusters, each of which represented part of the observed composite footprint (Figure 6a). These clusters were much less frequently detected across repeated iterations of clustering in untreated LNCaP cells. To quantify the degree to which these three patterns were present in LNCaP-induced cells compared with untreated cells, we examined the correlation between cluster centers obtained by performing k-means clustering 100 times for induced and uninduced LNCaP DNase-seq data. Specifically, the correlation of each cluster center to the cluster centers from all previous iterations was computed. Correlations tightly distributed around 1.0 represent highly reproducible clusters across different runs, suggesting that the three patterns are robust and consistently observed at AR motifs. Correlations loosely distributed about values less than 1.0 indicate that the three DNase-seq patterns at AR motif matches are less reproducible. We found this correlation distribution to be significantly higher (Mann-Whitney *P *< 2.2e^-16^) for LNCaP-induced cells (Figure 6b), with the most robust clustering associated with AR binding (Figure S4B in Additional file 1) (Mann-Whitney *P *< 0.001 between each column of correlations). Increasing the value of k consistently identified the same three general patterns in LNCaP-induced DNase-seq data within the AR motif, with multiple clusters aggregating to each general pattern (Figure S4E in Additional file 1). Using correlation analysis to analyze clusters from different values of k revealed that k = 3 is the most appropriate value (Figure S4F in Additional file 1), supporting that three distinct patterns of DNase I cleavage exist within AR motifs. Overall, the three distinct patterns of DNase I protection appeared to be a robust phenomenon more often detected in LNCaP-induced DNase-seq data, suggesting that AR activation stabilizes specific chromatin structure around AR motifs.

AR binding has been associated with enrichment of palindromic full-site AR motifs (such as depicted in Figure 4a) as well as half-site motifs [43,44]. The directional footprinting in clusters 1 and 2 is indicative of only half of the full canonical AR motif being protected from DNase I cleavage, whereas cluster 3 is consistent with full-site protection. Our ability to detect this indicates that specific half-site usage is consistent across the entire population of cells, and does not fluctuate randomly. The spike in the center of cluster 3 corresponds to the degenerate bases in the middle of the AR motif, indicating reduced DNA protection between AR proteins within the dimer. A recent report examining the dynamics of AR dimerization showed, in an exogenous system, that the AR binding enhancer element of *TMPRSS2 *requires an AR dimer. Consistently, we observed a DNase-seq digestion pattern similar to that shown in cluster 3 within this enhancer element (Figure S4D in Additional file 1).

While we posited that full-site protection might reflect a stronger AR-DNA association, AR ChIP-seq peak scores were evenly distributed between the three clusters, suggesting similar binding strength (Figure S4C in Additional file 1). We next explored if each cluster exhibited different co-factor motif enrichment by *de novo *motif analysis of the 25 base pairs upstream and downstream of the motif clusters (Figure 6c). Within these intervals, we detected more significant enrichment of FOX family motifs in the highly protected portions (dips) of clusters 1 and 2. A motif consistent with NF1C, which was also detected in an analysis of ΔDNase regions (Table S3 in Additional file 1), was enriched only upstream of cluster 3. These analyses suggest that the two well-defined dips observed around the composite footprint (Figure 4a) correspond to FOX factor-mediated DNA protection, which is seen to a more noticeable degree in LNCaP-induced DNase-seq data. Overall, our footprinting analysis revealed three different stable modes of DNase-seq protection with AR binding that represent two phenomena: full- or half-site protection at full-site DNA motifs.

## Discussion

The AR is a transcription factor and a primary driver of prostate cancer. Understanding the key determinants of its transcriptional specificity remains a critical issue. By integrating analysis of DNase-seq data with AR ChIP-seq and mRNA-seq, we showed that AR activation induced genome-wide changes in chromatin structure that were associated with AR binding and transcriptional response. We also uncovered multiple modes of AR utilization of its DNA recognition motif. Although a subset of AR binding occurs in qualitatively poised chromatin exhibiting nucleosome depletion prior to hormone treatment, we demonstrated that AR binding is consistently associated with a quantitatively significant increase in DNase-seq signal, suggesting stabilization of nucleosome depletion and chromatin remodeling.

Several prior reports also support AR-induced chromatin remodeling [16,17], including a very recently published study utilizing DNase-seq by He *et al. *[30]. Our data combined with these prior reports suggest a different model for nuclear receptor interaction with the genome than that proposed by John *et al. *for the GR [29], where almost all GR binding occurred in poised DHS sites. The AR and GR, though possessing similar DNA-response elements, seem to display fundamentally different interactions with chromatin and DNA. Our data represent a significant additional resource for understanding the association between chromatin accessibility and nuclear receptor function for several reasons. First, our DNase-seq experiments were sequenced very deeply (approximately 130 million reads), which is similar to the depth of sequencing with which John *et al. *observed GR binding to poised chromatin. Second, we utilized a different AR ligand (R1881) and time point of 12 hours as compared with 4 hours by He *et al. *and 1 hour by John *et al. *Similar to He *et al.*, who also utilized a quantitative measure of change in DNase-seq signal, we observed that less than half of AR binding targets poised chromatin and we were also able to associate AR-induced chromatin remodeling with AR-induced transcriptional changes, suggesting that the mechanism of chromatin remodeling and its phenotypically relevant association with differential transcription requires longer periods of receptor activation. Importantly, we used a different statistical measurement of quantitative change in DNase-seq signal to reach the same result and conclusion. In our study and those by He *et al. *and John *et al.*, we note that the degree of nuclear receptor binding within regions of poised chromatin decreases with increased hormone treatment time (37% in He *et al.*, 88% in John *et al. *and 20% to 30% in our study). Although this observation is confounded by differences in receptor, receptor ligand, sequencing depth and DNase-seq protocol among the mentioned studies, these data suggest that more extensive comparative analyses over a full time course of ligand stimulation of both AR and GR are needed to fully understand the similarities and differences of different hormone receptors with respect to their interaction with chromatin.

While the majority of high confidence AR binding occurred in regions sensitive to DNase I cleavage, a substantial proportion of AR binding events occurred in regions of low DNase-seq signal. It is possible that inconsistent and/or intermittent nucleosome depletion at these genomic regions decreases DNA accessibility and limits detection by our assay; this attribute of nucleosome depletion appears to be associated with a slightly different AR motif. Consistently, we also found that AR binding (as measured by AR ChIP-seq signal intensity) is significantly lower in non-DHS regions than in DHS regions. Thus, it is plausible that regions that are identified with weaker AR binding and lower DNase-seq signal may experience a dynamic equilibrium of nucleosome and nuclear receptor binding, as has been previously proposed [14]. Loci with reduced DNase I cleavage and AR binding could reflect low levels of AR binding at linker regions of non-displaced nucleosomes or residual nucleosome occupancy, limiting accessibility to DNase I cleavage in the cell population.

AR footprinting analysis further revealed the complexity of the AR-DNA interaction. The aggregate DNase-seq signal around AR motifs demonstrated a relatively weak but consistent pattern of protection that corresponds to the expected binding pattern, consistent with other DNase I footprinting studies [26]. In addition, we found three distinct patterns of DNase I protection significantly associated with LNCaP cells treated with androgen. The footprint patterns suggest that either AR binds to the full AR consensus motif as a dimer (cluster 3) or only binds to half of the motif (clusters 1 and 2). We also cannot exclude the possibility that clusters 1 and 2 represent AR dimers with only one AR molecule binding to half of the consensus motif. AR binding to either half site did not appear to be random, as evidenced by reproducible detection of distinct clusters. In other words, random binding to either half site in a population of cells would not show consistent half-site protection. Intriguingly, clusters 1 and 2 may provide the first *in vivo *and endogenous evidence of functional AR monomers that have been suggested to exist as a stable subpopulation of AR molecules [45]. Only the AR binding sites that displayed a full-site dimer protection pattern (cluster 3) were enriched for the NF1C motif, which is a known co-factor of AR. Therefore, there appears to be multiple modes that AR binds to canonical DNA motifs *in vivo*, and these modes are associated with different co-factors. These observations are consistent with a recently proposed model of a transient interaction between nuclear receptors such as the AR and DNA rather than a stronger and more stable AR-DNA interaction [46]. Our analysis also provides the first evidence of substructure within a nuclear receptor footprint

The dynamics of AR-DNA binding are likely impacted by additional co-factors that may facilitate AR binding directly or indirectly. Distal regulatory elements identified by DNase-seq displayed an enrichment of SP1 and E2A/TCF3 motifs within DHS specifically accessible in LNCaP cells compared with 113 independent cell lines. TCF3, a basic helix loop helix factor involved in Wnt/β-catenin signaling [47,48], represents a new putative co-factor for the AR that warrants further investigation to understand its role in AR-mediated chromatin dynamics as well as the crosstalk between AR and β-catenin signaling. SP1 is especially interesting both because its motif was enriched in ΔDNase regions and also in light of a recent report that identified SP1 as necessary for the expression of a variety of chromatin modifying enzymes, such as the histone deacetylases 1 to 4 in LNCaP cells [49]. Additionally, small molecule inhibitors of histone deacetylases have been shown to decrease the growth rate of AR-positive prostate cancer cell lines [50,51] and disrupt AR-induced expression of its target genes [52]. Our relative enrichment score of less than one for the SP1 motif and an observation that SP1 motifs often co-localize with AR binding suggest complexity in the interplay between SP1 and the AR.

## Conclusions

Overall, these lines of evidence combined with our results warrant further investigation of SP1 in the context of AR binding and AR-induced chromatin remodeling. Our analyses show that qualitative and quantitative assessment of chromatin accessibility by DNase-seq is an important and useful tool for elucidating AR biology in prostate cancer cell line models.

## Materials and methods

### Cell culture

LNCaP cells were obtained from ATCC and maintained according to manufacturer instructions. Prior to cell treatment with either 1 nM R1881 (methyltrienolone) or vehicle (ethanol), cells were grown in Roswell Park Memorial Institute-1640 media containing 10% charcoal-dextran stripped serum for 60 hours.

### DNase-seq library generation and analysis

DNase-seq was performed as previously described [24,53]. Briefly, 10 × 10^6 ^cells were harvested for each condition (± androgen). Nuclei were extracted and digested with optimal concentrations of DNase I enzyme. After confirmation of adequate digestion, DNase I-digested ends were blunt ended, and a biotinylated linker was ligated to these ends. Fragments with linker attached were isolated, digested with MmeI, and captured using streptavidin-conjugated magnetic beads. A second linker was ligated to the MmeI-digested end, and then the fragments were amplified and subsequently purified via gel electrophoresis. These sequencing libraries were sequenced on the Illumina GAIIx sequencing platform (Illumina, San Diego, CA, USA). Three biological replicates were processed for each cell growth condition. Sequencing results were aligned to the human reference genome (NCBI Build 37) using the Burrows-Wheeler aligner (BWA)[54]. Alignments were filtered to remove problematic repetitive regions such as alpha satellites and PCR artifacts characterized by many sequences mapped to small genomic locations. Biological replicates were compared for reproducibility, and then combined. In our cross-replicate analysis, we determined one of three biological replicates of LNCaP-induced DNase-seq to be discordant from the other two biological replicates and thus removed that replicate from the combined DNase-seq sequence set. The final base-pair resolution signal to reflect chromatin accessibility was generated using F-seq [31]. Discrete peaks were called by fitting DNase-seq signal data to a gamma distribution and then determining the signal value that corresponded to *P *< 0.05. Gene-relative categories were defined as previously described [24].

### Identification of increases and decreases in DNase-seq signal

To determine regions of significant change in DNase-seq signal with androgen induction, we used the edgeR bioconductor package [32,55]. The edgeR package is designed to detect differences in count data among groups of samples containing biological and technical replicates. Prior to running the algorithm, we defined windows in which to compare DNase-seq signal across replicates by first taking the union set of all identified DHS sites in both LNCaP and LNCaP-induced cells.

This approach allows for inclusion of regions that contain an increase or decrease in DNase-seq signal such that they cross the threshold defining a DHS site. The defined union set was then divided into overlapping windows of 300 bp. DHS regions smaller than the window size were expanded to the window size. Regions larger than the window size were tiled with overlapping windows, where the overlap varies depending on the size of the hypersensitive region to tile. We start by finding the number of windows that would fit completely inside the defined DHS site using the default overlap. If these windows discard fewer than 10% of the bases on each edge of the DHS site, we tile the site using these windows. If using the default overlap would cause us to lose more than this edge threshold, we add another window and adjust the overlap so that the windows exactly cover the entire DHS region. We find that these windows cover almost all of the DHS bases in the original, while minimizing the number of non-DHS bases considered for the downstream analysis. Our approach created approximately 550,000 windows for differential analysis among five replicates (three LNCaP, two LNCaP-induced). The number of tags mapping to each window in each replicate were extracted, and regions with a sum total of less than five reads were eliminated. We then used edgeR to call windows with significantly different counts in each pairwise comparison at two thresholds: strict (FDR < 0.05) and loose (unadjusted *P *< 0.05). Finally, neighboring windows that were identified as having a significantly higher DNase-seq signal in a condition were merged. To generate a normalized differential tag count for regions, the number of DNase-seq tags within each LNCaP and LNCaP-induced DHS region was determined and normalized to the average number of tags in either all LNCaP or LNCaP-induced DHS site. For each of the 175,796 union DHS regions, the normalized number of tags in LNCaP in the region was subtracted from the normalized number of tags in LNCaP-induced to give the differential tag score for each region.

### RNA expression analysis

RNA expression in response to androgen induction in LNCaP was analyzed using both exon microarrays and mRNA-seq. Total RNA was extracted using TRIzol (Sigma-Aldrich, St. Louis, MO, USA) from the same cell growth as used for DNase-seq and hybridized to Affymetrix Exon 1.0 ST arrays (Affymetrix, Santa Clara, CA, USA) using a standard protocol. Resulting .CEL files were summarized into expression measures at the gene-core level using Affymetrix Power Tools and Robust Multi-array Average (RMA) normalization [56]. Differential expression upon androgen induction was determined using the Statistical Analysis of Microarrays bioconductor package [57]. Two biological replicates were processed for exon array analysis.

RNA for use in mRNA-seq was isolated from three independent growths of LNCaP with or without androgen using the Ambion miRVANA miRNA isolation kit (Invitrogen, Grand Island, NY, USA). Induction of canonical AR target gene expression was confirmed by qPCR, and RNA quality was verified using an Agilent Bioanalyzer (Agilent Technologies, Santa Clara, CA, USA). All RNA used for subsequent library preparation had an RNA integrity number greater than 9.0. mRNA-seq libraries were created using the Illumina mRNA-seq protocol and kit then sequenced on the Illumina GAIIx platform. Resulting sequence data was aligned to the human reference genome (NCBI Build 37) first using BWA [54]. Reads unaligned by BWA were independently aligned with TopHat [58] to only known and annotated splice junctions. The results combined and filtered to remove non-unique reads. Technical replicates were merged such that three biological replicates (± androgen) were available for subsequent analysis. The reads per kilobase mapped expression measure was computed for each RefSeq gene model in each replicate, leaving out tags mapping to the 3' untranslated region of genes [59]. To identify RefSeq genes differentially expressed between LNCaP and LNCaP-induced cells, we first counted the number of mRNA-seq tags that fell within exons of RefSeq gene models in each biological replicate, resulting in a tag count value for each gene. We then used the edgeR bioconductor package to detect genes whose expression differed with AR activation, FDR < 0.05.

### Correlation of ΔDNase increases and decreases with expression increases and decreases

To establish the relationship between differential chromatin and differential expression, we tested for significance in overlap in both directions, that is, we tested if ΔDNase regions tend to be located near differentially expressed genes, and then tested if differentially expressed genes tend to have a ΔDNase region nearby. We first assigned each DHS site to its nearest gene and intersected these nearest genes with each AR-regulated gene set (AR mRNA-seq upregulated, AR mRNA-seq downregulated, and four sets from Massie *et al. *[40]: Massie early up, Massie early down, Massie late up, Massie late down). We calculated the significance of the ΔDNase association to differentially regulated genes by permuting the set of all RefSeq genes 100,000 times, randomly selecting the number of genes for each comparison, and intersecting those random sets with the genes related to AR-induced expression changes. This established a null distribution of overlaps in random intersects. We also conducted the same analysis in the opposite direction to relate expression change ΔDNase regions. Using the University of California Santa Cruz (UCSC) Known Genes table, we merged all isoform coordinates for each gene and found all ΔDNase increase or decrease sites within a surrounding 20 kb window. We calculated pairwise overlaps of ΔDNase sites between each ΔDNase increase or decrease list and these lists of all nearby ΔDNase sites. If a gene contained a ΔDNase site within 20 kb, it was counted as a match. We then permuted genes located all nearby DHS sites 1,000 times, and tested for overlap to create a null distribution of overlap count.

### Androgen receptor ChIP-seq

AR ChIP-seq data was obtained from accession numbers [GSE14097] and [GSE28126] through the NCBI Gene Expression Omnibus portal. [GSE28126] was recently published [40] as part of a study identifying AR ChIP-seq peaks in LNCaP cells after 4 hours of 1 nM R1881 stimulation. [GSE14097] [39] contains AR ChIP-seq data from LNCaP cells treated with either 10 nM R1881 for 16 hours or vehicle for the same length of time. Coetzee AR ChIP-seq was conducted after 4 hours of either 10 nM dihydrotestosterone or ethanol treatment of LNCaP cells [17,41]. Raw sequence files were processed through the same pipeline as our DNase-seq data [31] to obtain aligned sequences. Model-based analysis for ChIP-seq (MACS) [60] was used with default parameters to identify regions of significant AR ChIP enrichment in LNCaP-induced relative to LNCaP. To generate the common AR peaks list, we intersected the peak calls from the two data sets, considering peaks to be overlapping if they intersect by at least one base pair.

### Self-organizing maps

To identify DHS sites specific to our two cell types, we used a SOM built from DNase-seq data generated by our group from 113 lines [61]. SOMs are artificial neural networks that learn patterns in data by iteratively assigning data points to cluster centers. The SOM eventually assigns each DHS site to a cluster with the most similar hypersensitivity profile. We are using SOMs to characterize DNase I hypersensitivity profiles across over 100 cell lines (NS, in preparation). For this analysis, we were interested in clusters specific to LNCaP cell lines. We first built a data matrix by counting the number of reads mapping in each peak region in each cell type. We quantile-normalized the scores by cell type and then capped them at the 99th quantile (by setting the top 1% of scores to a maximum value), and then row-scaled the scores to a decimal between 0 and 1. After normalization, capping and scaling, we built an SOM using the Kohonen package in R. This SOM used a hexagonal 50 × 50 grid (for 2,500 total nodes). We then took each node and selected the 50 regions closest in distance to the node center, and submitted these to MEME for *de novo *motif analysis [62]. We then matched these motifs to publicly available DNA binding motifs in TRANSFAC 7.0 [63] and JASPAR 2010 [64] using STAMP [65].

### Motif analysis

To determine motif enrichment in regions of interest we utilized used three algorithms: MEME, cERMIT [66], and CentDist [67]. MEME and cERMIT report identified DNA motifs not matched to known motifs. If MEME was run on regions falling within DHS sites (all but analysis in Figure 3e), we used a first order background model common to DHS sites. cERMIT was run using ΔDNase *P*-value as evidence for directing motif analysis. CentDist identifies motifs enriched within a region and ranks them relative to their distribution within each region, reporting publicly available motifs that are found in regions. Motifs identified in Figures 2 and 3 were identified in at least two of these algorithms. If a motif was reported as enriched by MEME and cERMIT, it was included in our results if its match to publicly known motifs, determined by STAMP or TomTom (using JASPAR and Uniprobe databases) [68], was significant (E < 0.05). Results from CentDist are shown in Table S3 in Additional file 1.

### Androgen receptor footprint analysis

To generate an aggregate plot of DNase-seq signal around AR motifs, we scanned DHS regions containing AR binding sites using a first order log likelihood scanner with a slightly modified AR position weight matrix (PWM) from the JASPAR database. The MA0007.1 matrix was trimmed by discarding low information base pair positions surrounding positions 4 to 18, resulting in a 15 bp PWM. PWM motif scores that scored lower than the 90^th ^percentile of all match scores were discarded, and the strand with the stronger PWM match was chosen if both strands at a location matched the PWM within the 90^th ^percentile or higher. DNase-seq reads mapping to each base at the motif site and surrounding 100 bp were collected and the sum of each position was calculated.

For cluster correlation analysis, the k-means algorithm [69] was run 100 times to yield k × 100 cluster centers, where each cluster center is a vector of values of length 31 (clustering was performed on DNase-seq signal mapping to 15 bp on both sides of center of AR motif). Cluster centers from one run *i *to all other runs *(1, ..., i-1, i+1, ..., 100) *were compared. Each cluster center from a single run was matched to another cluster center in another run in a pairwise manner that identifies maximum correlation; this procedure was performed across all pairs of runs to assess the similarity and reproducibility of results over multiple runs of the algorithm.

For aggregate visualization of clusters, we tabulated DNase-seq tag counts 100 bp around AR PWM matches classified into each cluster within 'R1881 intersect' ChIP peaks that were DHS in both LNCaP-induced and LNCaP. MEME was used to search for *de novo *motifs 25 bp up- and downstream from PWMs classified into each cluster. TomTom was used to match significant motif matches to publicly available motifs (E < 0.05).

### Data access

DNase-seq data from this study can be visualized using the UCSC Genome Browser [70]. Specifically, click on the 'Genome Browser' option then click on the 'configure tracks and display' button. Under the section entitled 'Regulation', find the subsection 'ENC DNase/FAIRE' for ENCODE Open Chromatin by DNase I HS and FAIRE, and then click on the 'Duke DNaseI HS' link. In the menu of cell lines that will appear, click on the two boxes in the row labeled LNCaP (one for 'no treatment' and one for 'methyltrienelone (androgen)'), and this will allow for data visualization. Processed and raw DNase-seq data has also been deposited through the NCBI GEO website under accession number [GSE32970]. Within that accession number, data for LNCaP is available under [GSM816637] (Duke_DnaseSeq_LNCaP), and data for LNCaP-induced is available under [GSM816634] (Duke_DnaseSeq_LNCaP_androgen). Exon array expression data is publicly available through the NCBI GEO website under accession number GSE15805. Within this accession number, the two LNCaP replicates are under GSM443919 and GSM443920, and the two LNCaP-induced replicates are under GSM443921 and GSM443922. mRNA-seq data from this study is available through the NCBI GEO website under accession number GSE34780.

## Abbreviations

AR: androgen receptor; bp: base pairs; BWA: Burrows-Wheeler Aligner; ChIP-seq: chromatin immunoprecipitation coupled with high-throughput sequencing; CTCF: CCCTC-binding factor; DHS: DNase I hypersensitive; DNase-seq: DNase I hypersensitivity analysis coupled with high-throughput sequencing; FDR: false discovery rate; FOX: Forkhead box; FAIRE: formaldehyde-assisted isolation of regulatory elements; GR: glucocorticoid receptor; kb: kilobase pairs; mRNA-seq: messenger RNA abundance measured by high-throughput sequencing; NF1C: nuclear factor 1 C-type; PCR: polymerase chain reaction; PWM: position weight matrix; qPCR: quantitative polymerase chain reaction; RMA: Robust Multi-array Average; SOM: self-organizing map.

## Competing interests

The authors declare that they have no competing interests.

## Authors' contributions

AKT, TSF, GEC and PGF designed the experiments. AKT and LS performed the experiments. GAC contributed AR ChIP-seq data and revised the manuscript. AKT, GGY, YS, NCS, BST, SGG and UO analyzed the data. AKT, TSF, GEC and PGF wrote the manuscript. All authors revised the manuscript and approved the final version.

## Supplementary Material

Additional file 1 **Supplemental information containing four supplemental figures and three supplemental tables**. Click here for file

Additional file 2 **Table S4 detailing RefSeq genes identified as differentially expressed in response to androgen receptor activation by mRNA-seq**. mRNA from three biological replicates of LNCaP and three biological replicates of LNCaP-induced was extracted and sequenced. Genes differentially regulated by AR activation were determined using edgeR at a threshold of FDR < 0.05. Click here for file

Additional file 3 **Table S5 showing a comparison of androgen receptor-regulated genes as determined by mRNA-seq to previously published androgen receptor-regulated gene lists**. Genes identified as differentially regulated by AR activation are shown along with all other RefSeq genes, ranked in order by the number of published studies that identify the gene as AR-regulated. Click here for file
