# Supplementary material for: Chromatin accessibility reveals insights into androgen receptor activation and transcriptional specificity
Source: Genome Biol. 2012 Oct 3;13(10):R88. doi: 10.1186/gb-2012-13-10-r88 (PMC3491416; doi:10.1186/gb-2012-13-10-r88)
Supplement: Additional file 1 — Supplemental information containing four supplemental figures and three supplemental tables. [file gb-2012-13-10-r88-S1.pdf]

Supplemental Information

Table S1: Summary of DNase-seq experiments. Three biological replicates of LNCaP and two biological replicates of LNCaP Induced were combined to create final DNase-seq libraries.

	**LNCaP**	**LNCaP Induced**
**Total DNase-seq Reads**	129,131,592	138,464,636
**Number of DHS**	144,070	140,966
**Bases within DHS**	86,989,168	82,887,882
**Percentage of genome**	3.01	2.87

Table S2: Selected motifs enriched within ΔDNase regions out of the top 50 significant motifs detected for each set by CentDist.

**Strict ΔDNase Increase**			
**Rank**	**Name**	**Logo**	**Score**
1	V$AR_01	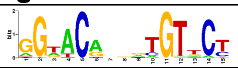	53.32
3	V$jaspar_Foxa2	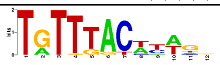	34.38
4	V$HNF3ALPHA_Q6	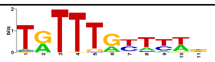	33.55
5	V$jaspar_NFIC	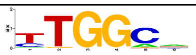	17.66
15	V$AP1_Q4	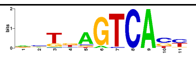	7.77
34	V$GATA1_04	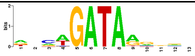	5.73
36	V$jaspar_TAL1__TCF3	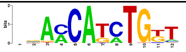	5.59
39	V$jaspar_YY1	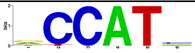	5.31
41	V$OCT1_Q6	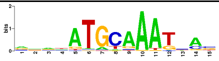	5.14
50	V$NKX3A_01	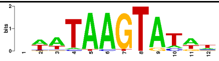	4.64
**Loose ΔDNase Increase**			
1	V$AR_01	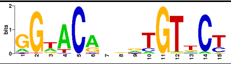	82.99
2	V$HNF3ALPHA_Q6	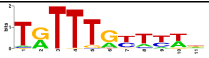	69.53
3	V$DBP_Q6	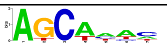	28.91
4	V$NF1_Q6	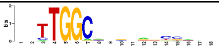	28.83
5	V$AP1_C	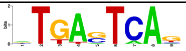	26.91

11	V$MAF_Q6_01	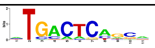	19.52
30	V$OCT1_B	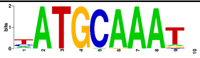	10.29
33	V$STAT_01	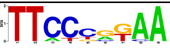	9.99
38	V$GATA1_04	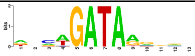	9.67
42	V$PBX1_01	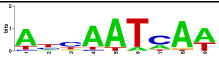	9.07
**Loose ΔDNase Decrease**			
1	V$AP2_Q6_01	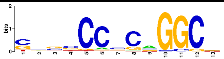	11.86
3	V$jaspar_PLAG1	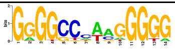	11.25
10	V$KROX_Q6	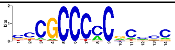	8.79
13	V$jaspar_NFKB1	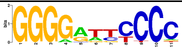	8.16
23	V$AP4_Q6_01	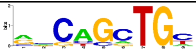	5.99

Table S3: CentDist motif analysis for ΔDNase regions with and without AR binding as well as AR binding regions with minimal DNase-seq signal.

**Strict ΔDNase Increase with AR binding peak**			
**Rank**	**Name**	**Logo**	**Score**
1	V$AR_01	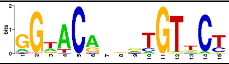	49.00
3	V$HNF3ALPHA_Q6	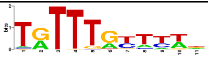	25.18
5	V$DBP_Q6	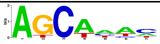	14.50
6	V$jaspar_NFIC	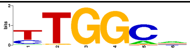	13.34
17	V$jaspar_AP1	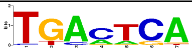	6.45
**Strict ΔDNase Increase with no AR binding peak**			
2	V$HNF3ALPHA_Q6	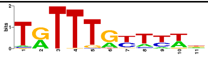	24.18
3	V$AR_01	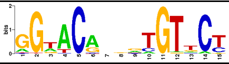	22.94
5	V$jaspar_NFIC	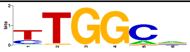	11.77
8	V$AP1_01	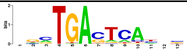	7.86

9	V$jaspar_SRY	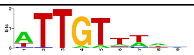	7.41
**Loose ΔDNase Increase with AR binding peak**			
1	V$AR_01	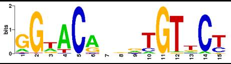	79.01
3	V$HNF3ALPHA_Q6	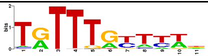	50.31
4	V$jaspar_FOXA1	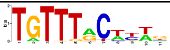	50.03
5	V$jaspar_NFIC	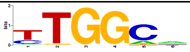	27.85
6	V$DBP_Q6	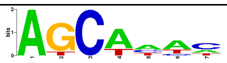	21.62
**Loose ΔDNase Increase with no AR binding peak**			
1	V$jaspar_CTCF	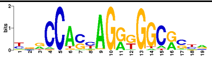	55.92
2	V$jaspar_FOXA1	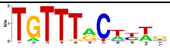	54.39
4	V$AR_01	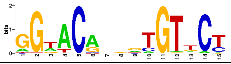	42.12
6	V$jaspar_NFIC	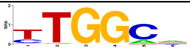	28.52
7	V$AP1_C	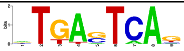	24.19
**AR binding, negligible DNase-seq signal**			
1	V$AR_01	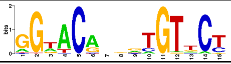	30.87
2	V$HNF3ALPHA_Q6	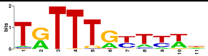	15.42
3	V$GATA6_01	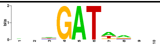	5.19
4	V$DBP_Q6	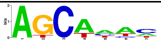	5.18
7	V$NRF2_Q4	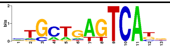	4.32

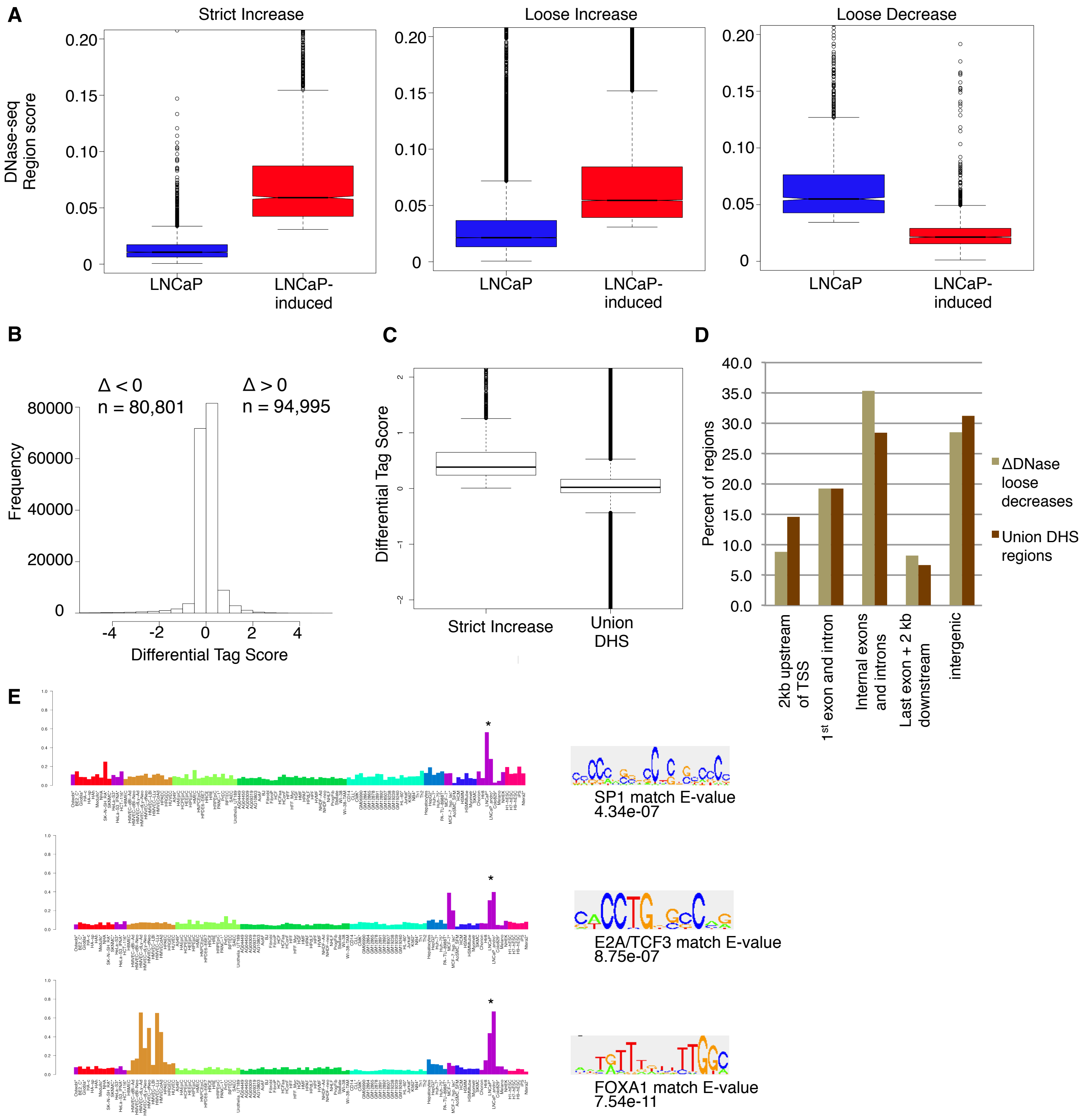

A
Strict Increase
Loose Increase
Loose Decrease
DNase-seq Region score
0.20
0.15
0.10
0.05
0
LNCaP
LNCaP-induced
B
Δ < 0
n = 80,801
Δ > 0
n = 94,995
Frequency
80000
60000
40000
20000
0
-4
-2
0
2
4
Differential Tag Score
C
Differential Tag Score
Strict Increase
Union DHS
D
Percent of regions
40.0
35.0
30.0
25.0
20.0
15.0
10.0
5.0
0.0
ΔDNase loose decreases
Union DHS regions
2kb upstream of TSS
1st exon and intron
Internal exons and introns
Last exon + 2 kb downstream
intergenic
E
SP1 match E-value 4.34e-07
E2A/TCF3 match E-value 8.75e-07
FOXA1 match E-value 7.54e-11

Figure S1: Confirmation of ΔDNase regions. (a) Using the maximum DNase-seq score identified for each ΔDNase region in LNCaP and LNCaP induced (for score explanation, see [8]), we show that strict and loose increase and decrease ΔDNase regions do indeed identify genomic regions with significantly different DNase-seq signal (Mann-Whitney p-value < 0.05). (b) Differential DNase-seq tag score frequency shown for the union of all DHS in LNCaP and LNCaP induced. The median differential tag scores is greater than zero, and more regions have a positive score than negative, validating the lack of strict ΔDNase decrease regions. (c) The differential tag score also validates that regions of strict increase ΔDNase possess significantly more DNase-seq signal (Mann-Whitney p-value < 0.05). (d) Distribution of loose decrease ΔDNase regions relative to all union DHS regions shows a similar trend of changes in DNase-seq signal favoring intergenic and intragenic regions rather than the promoter. (e) Three self-organizing map results. Shown is the relative chromatin accessibility plot, with cell lines labeled along the x-axis and DNase-seq score on the y-axis. Asterisks mark LNCaP and LNCaP-induced data. Also shown is the enriched motif detected for each result, and the calculated E-value of the best match to the indicated known DNA binding motif.

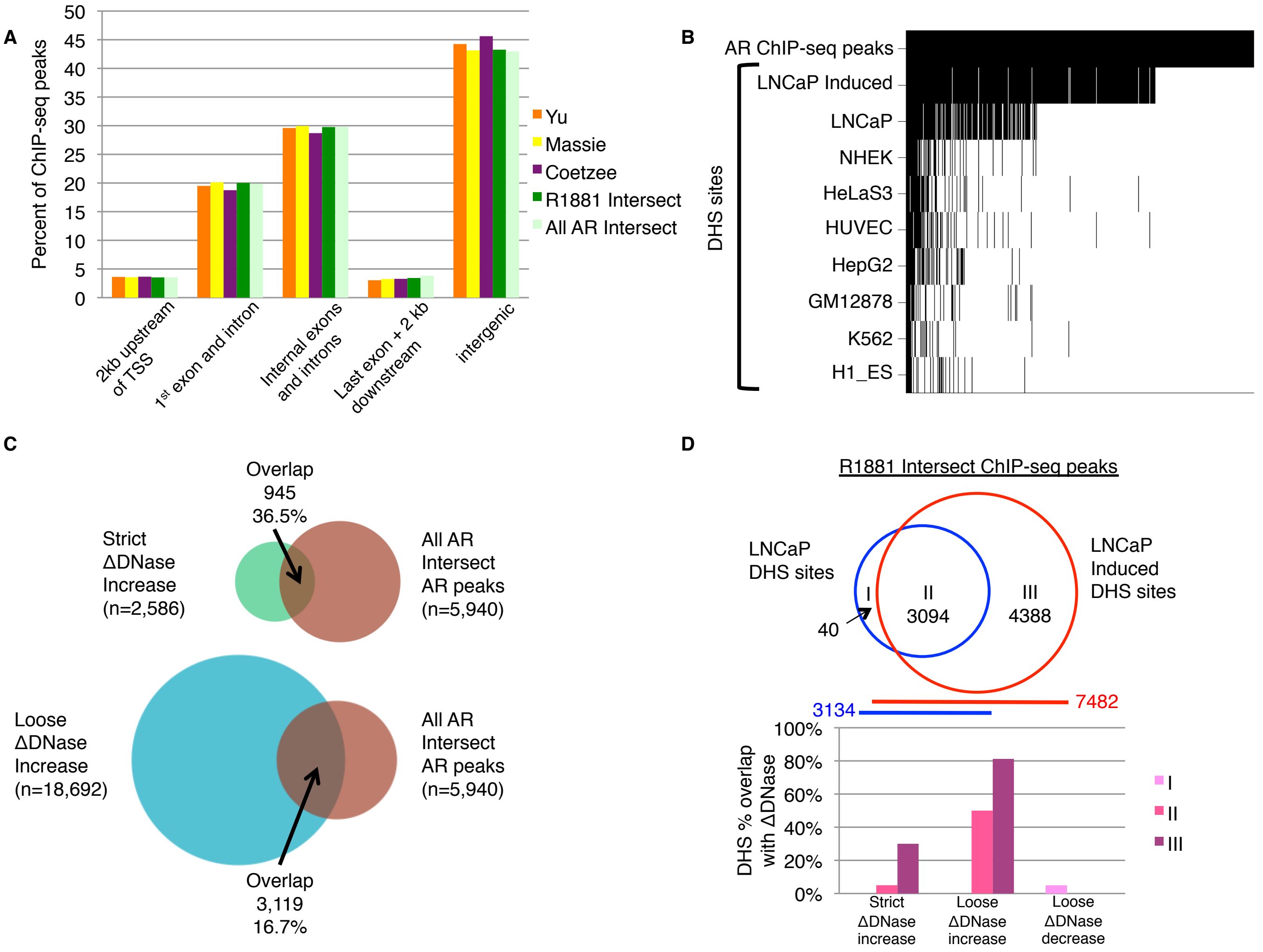

Figure S2: Characteristics of different AR ChIP-seq data sets in relation to DHS and ΔDNase regions. (a) Distribution of AR ChIP-seq peaks from each study and the two combined data sets relative to genic elements. (b) A heatmap of overlap between "All AR intersect" AR binding sites and DHS sites from LNCaP, LNCaP induced, and seven other cell lines indicating that AR binding preferentially occurs in LNCaP/LNCaP induced DHS. c) Comparison of ΔDNase regions to All AR intersect AR binding sites indicating that a substantial proportion of these ΔDNase regions contain a high confidence AR binding site. (d) Figure 3d recreated for the R1881 intersect ChIP-seq peak set, confirming that both poised and LNCaP induced DHS only regions with an AR binding site are enriched for ΔDNase regions.

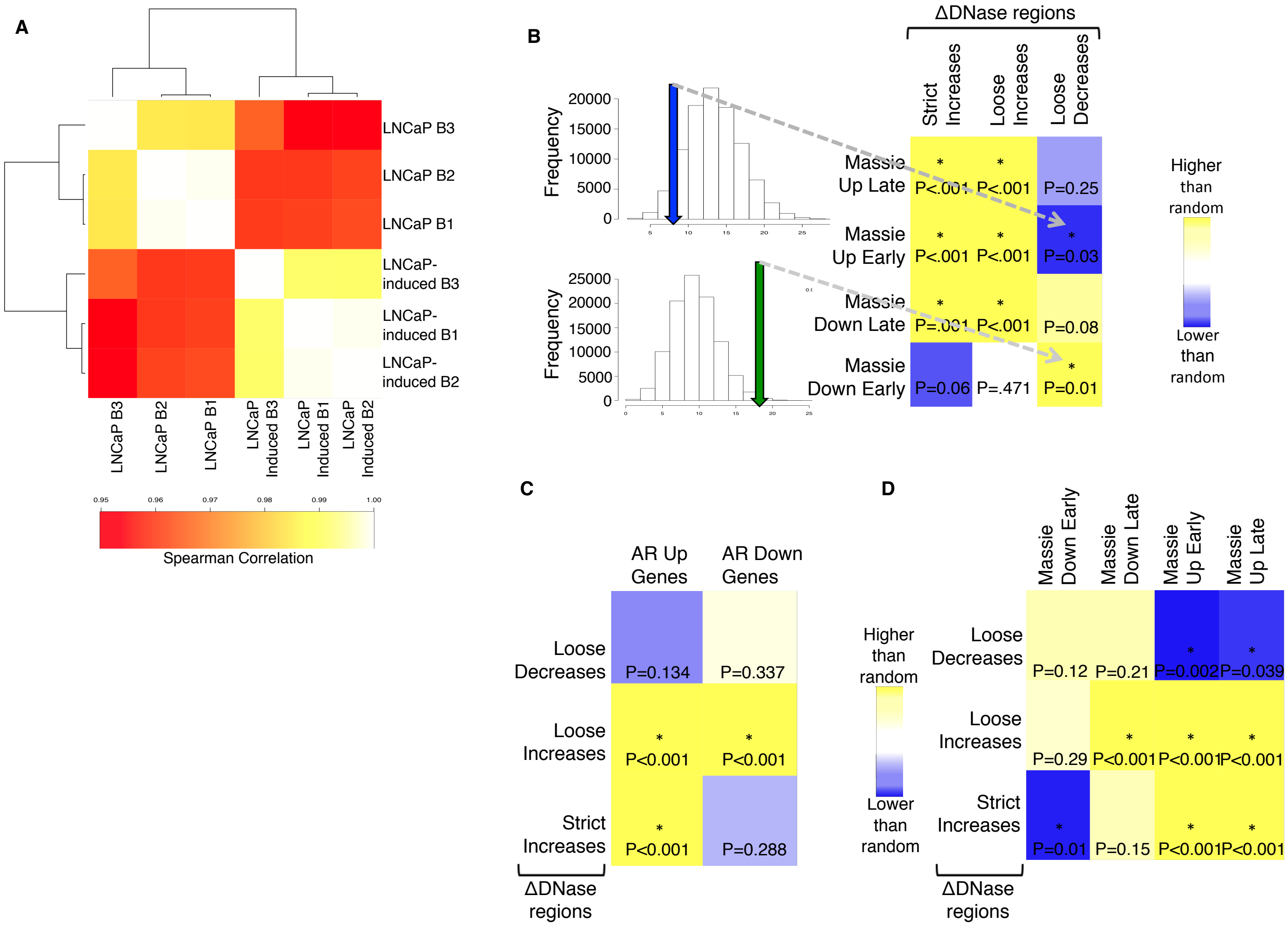

Figure S3: Enrichment of expression changes to ΔDNase regions. (a) Spearman correlation heatmap of expression (RPKM) data from each biological replicate for LNCaP and LNCaP induced. (b) ΔDNase changes permuted against genes up- and down-regulated at early (4 hr) and late (24 hr) time points according to Massie et al. [1] Similar to Figure 4, blue shading represents an absence of ΔDNase regions around regulated genes greater than expected by chance, whereas yellow shading represent an enrichment of ΔDNase regions around regulated genes greater than expected by chance. (c) Analysis of Figure 4B in the opposite direction, relating AR-regulated expression to ΔDNase regions within 20kb. Shown are permutation p-values and associations considered significant (asterisk). (d) Analysis of Supplemental Figure 3B in the opposite direction, relating AR-regulated expression from Massie et al. to ΔDNase regions Shown are permutation p-values and associations considered significant (asterisk).

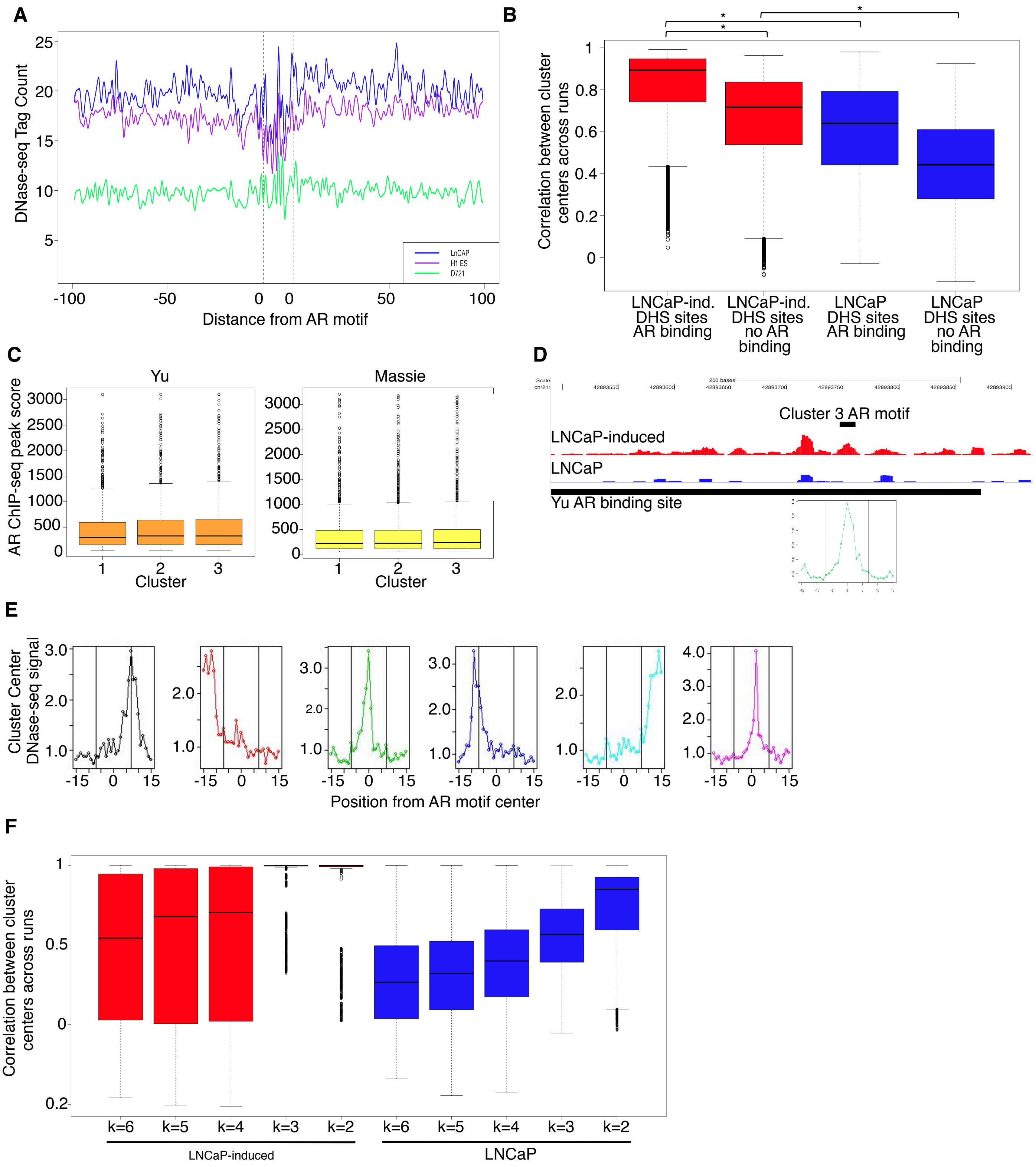
A
DNase-seq Tag Count
Distance from AR motif
LnCAP
H1 ES
D721
B
Correlation between cluster centers across runs
LNCaP-ind. DHS sites AR binding
LNCaP-ind. DHS sites no AR binding
LNCaP DHS sites AR binding
LNCaP DHS sites no AR binding
C
AR ChIP-seq peak score
Yu
Massie
Cluster
D
Cluster 3 AR motif
LNCaP-induced
LNCaP
Yu AR binding site
E
Cluster Center DNase-seq signal
Position from AR motif center
F
Correlation between cluster centers across runs
k=6
k=5
k=4
k=3
k=2
LNCaP-induced
LNCaP

Figure S4: DNaseI protection around AR motifs. (a) Aggregate plot of LNCaP (blue) and H1 embryonic stem cell (purple) and D721 medulloblastoma cell (green) DNase-seq signal around high scoring AR PWM matches within DHS sites in each cell line. Despite D721 and H1 ES cells lacking AR expression, a similar DNaseI protection patterns is seen in each cell line, suggesting another transcription factor may be utilizing this motif. (b) Correlation between cluster centers for 100 runs for sub-samples of 1000 regions containing AR motifs. The three clusters observed are most reproducible in LNCaP induced DHS sites that also have an AR binding site. AR activation to the nucleus also creates similar clusters, but to a statistically significant lesser degree. Also shown are the correlations across cluster runs for LNCaP DNase-seq data at the same AR binding and non-binding regions as analyzed for LNCaP induced data. Asteriks indicate significantly different correlation distributions (Mann-Whitney p-value < 0.05). (c) AR ChIP-seq peak score for the Massie and Yu AR binding peaks that contain a cluster 1, 2, or 3 AR PWM match. There is no statistical difference between the AR binding strength in each cluster. (d) Example of cluster 3 AR motif within the upstream AR binding enhancer of *TMPRSS2*. This enhancer was predicted to require a full AR dimer to function [2], consistent with a cluster 3 DNase-seq pattern. Y-axis represents number of DNaseI cuts at each base pair, and axes are fixed at 31 cuts for both LNCaP and LNCaP-induced rows. (e) Cluster centers from k-means clustering with k=6. Note that for clusters 2 and 5, the variation in signal detected falls outside the AR motif itself. (f) Correlation between cluster centers for 100 iteration of k-means clustering with various values of k for DNase-seq signal around AR motif matches. The strongest and most reproducible clusters were found for k=3.

Supplemental References

1. Massie CE, Lynch A, Ramos-Montoya A, Boren J, Stark R, Fazli L, Warren A, Scott H, Madhu B, Sharma N, et al: **The androgen receptor fuels prostate cancer by regulating central metabolism and biosynthesis.** *EMBO J* 2011, **30:**2719-2733.
2. van Royen ME, van Cappellen WA, de Vos C, Houtsmuller AB, Trapman J: **Stepwise androgen receptor dimerization.** *J Cell Sci* 2012.
